# A Review of the Receptor-Binding Properties of *p*-Synephrine as Related to Its Pharmacological Effects

**DOI:** 10.1155/2011/482973

**Published:** 2011-08-01

**Authors:** Sidney J. Stohs, Harry G. Preuss, Mohd Shara

**Affiliations:** ^1^School of Pharmacy and Health Professions, Creighton University Medical Center, Omaha, NE 68178, USA; ^2^Department of Medicine and Pathology, Georgetown University Medical Center, Washington, DC 22039, USA; ^3^Faculty of Pharmacy, Jordan University of Science and Technology, Irbid 22110, Jordan

## Abstract

Bitter orange (*Citrus aurantium*) extract and its primary protoalkaloid *p*-synephrine are used widely in weight loss/weight management and sports performance products. Because of structural similarities, the pharmacological effects of *p*-synephrine are widely assumed to be similar to those of ephedrine, *m*-synephrine (phenylephrine), and endogenous amine neurotransmitters as norepinephrine and epinephrine. However, small structural changes result in the receptor binding characteristics of these amines that are markedly different, providing a plausible explanation for the paucity of adverse effects associated with the wide-spread consumption of *p*-synephrine in the form of dietary supplements as well as in various *Citrus* foods and juices. This paper summarizes the adrenoreceptor binding characteristics of *p*-synephrine relative to *m*-synephrine, norepinephrine, and other amines as related to the observed pharmacological effects.

## 1. Introduction

**Figure 1:**
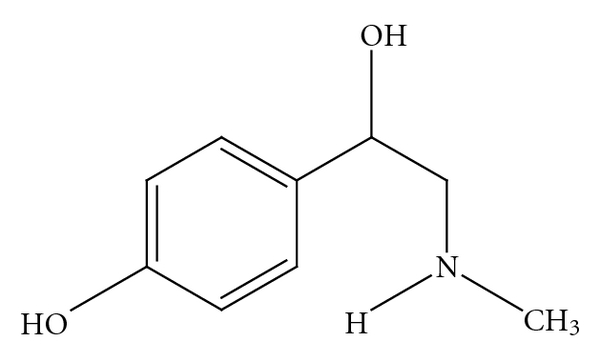
*p*-Synephrine.

*p-*synephrine (Figure 1) is the primary protoalkaloid in *Citrus aurantium *(bitter orange) and other *Citrus *species [1–14]. Bitter orange extract and *p*-synephrine are widely used in weight management and sports performance products. In addition to its consumption in dietary supplements, *p-*synephrine is daily consumed in various foods and juices derived from *Citrus* species as Seville oranges, mandarin oranges, clementines, Marrs sweet oranges, Nova tangerines, grapefruits and other orange-related species containing *p*-synephrine [12–15]. In spite of the wide-spread consumption and the lack of directly attributable adverse effects to *p*-synephrine and bitter orange extract [16–18], the safety of *p*-synephrine is frequently questioned. Various articles refer to the potential cardiovascular hazards that may occur as the result of using dietary supplements containing *p*-synephrine and bitter orange extract [19–24], with reference being made to clinical case reports that involve multiherbal and polyalkaloidal and poly-protoalkaloidal products [16, 17]. 

**Figure 2:**
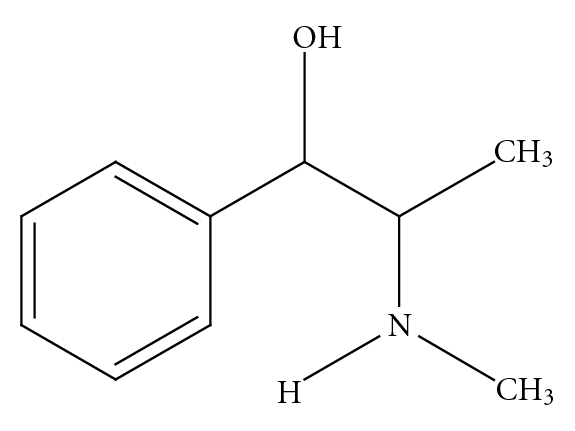
Ephedrine.

**Figure 3:**
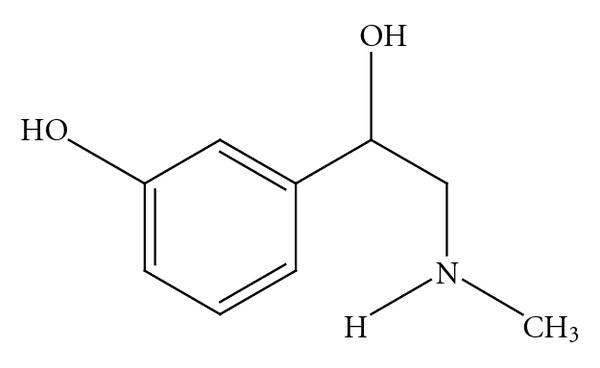
*m*-Synephrine.

Current confusion regarding the safety of bitter orange extract and *p*-synephrine is clouded by multiple issues, including the use of complex mixtures of ingredients in products that include bitter orange extract, the existence of some structural similarities with ephedrine (Figure 2), the misunderstandings regarding the isomeric forms of synephrine and their differing pharmacological properties [18], and the release of misleading information by governmental agencies [25]. The projected warnings regarding cardiovascular risks are extrapolated from studies involving *m*-synephrine (Figure 3) and ephedrine which are not constituents of bitter orange, extract. Furthermore, *m*-synephrine does not occur naturally in plants, including *Citrus* species [1–15]. Pellati and Benvenuti [26] summarized 19 chromatographic and electrophoretic analytical methods for synephrine protoalkaloids in bitter orange and in no case was *m*-synephrine detected.

*p*-synephrine (see Figure 1) is a phenylethanolamine derivative with the hydroxy group in the *para*position on the benzene ring of the molecule [3, 6, 8, 10], while *m*-synephrine (Figure 3), which is also known as phenylephrine, has a hydroxylgroup in the *meta*position on the benzene ring [10]. As shall be discussed, the location of the hydroxygroup on the ring greatly alters the receptor binding characteristics and as a consequence greatly alters the pharmacological properties.

**Figure 4:**
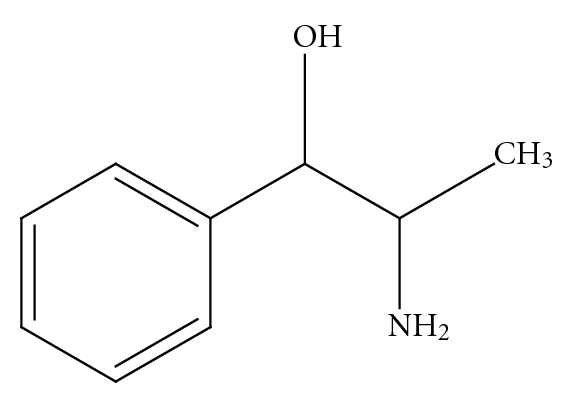
Phenylpropanolamine.

**Figure 5:**
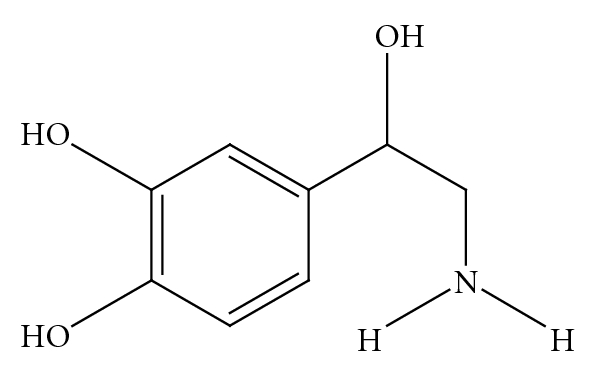
Nor-epinephrine (Nor-adrenaline).

**Figure 6:**
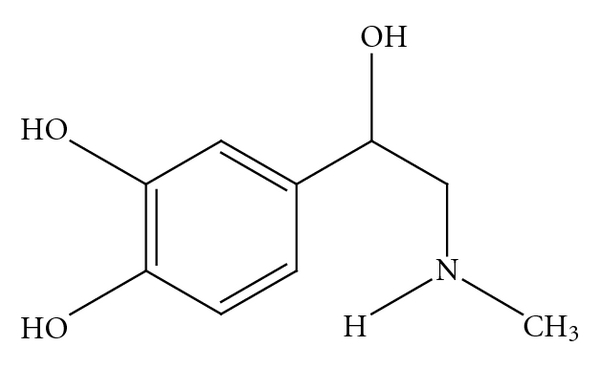
Epinephrine (Adrenaline).

**Figure 7:**
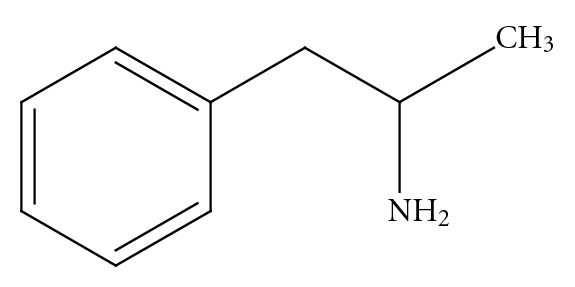
Amphetamine.

*p*-synephrine is structurally related to ephedrine [3, 6, 8] (Figure 2). However, ephedrine is a phenylpropanolamine (Figure 4) derivative and does not contain a *para*-substituted hydroxy group. The addition of the *para*hydroxygroup on the *p*-synephrine molecule, as well as the lack of the methyl group on the side chain change the stereochemistry and as a consequence the receptor binding characteristics and the pharmacokinetic properties, including the ability of *p*-synephrine to cross the blood-brain barrier. The lipid solubility of *p*-synephrine as compared to ephedrine is significantly decreased, resulting in little transport of *p-*synephrine into the CNS as compared to ephedrine [27]. As will be discussed, as a result of these structural differences, *p*-synephrine exhibits little or no CNS and cardiovascular stimulation as compared to ephedrine (Figure 2), norepinephrine (noradrenaline; Figure 5), epinephrine (adrenaline; Figure 6), *m-*synephrine (Figure 3), and amphetamine (Figure 7).

Most review articles regarding *p*-synephrine and bitter orange extract assume that because of the structural similarities, *p*-synephrine, *m*-synephrine, ephedrine, norepinephrine (Figure 5), epinephrine (Figure 6), and amphetamine (Figure 7) all exert similar properties and fail to consider or discuss the receptor binding studies that have been conducted [19–24]. This paper summarizes the studies that have been conducted to date on the adrenoreceptor binding properties of *p*-synephrine relative to *m*-synephrine (phenylephrine), norepinephrine, and other amines. The results provide a receptor-based foundation for explaining and interpreting the pharmacological effects as well as the paucity of toxicological effects of *p*-synephrine observed in human clinical as well as animal studies.

## 2. Receptor Binding Studies

In general, vasoconstriction is produced when ligands acting as agonists bind to *α*-adrenoreceptors, cardiovascular contractility and increased heart rate occur in response to *β*-1 adrenoreceptor binding, while bronchodilation occurs in response to *β*-2 adrenoreceptor binding [24]. As previously noted, it is widely assumed that *p*-synephrine exhibits mechanistic properties similar to those observed for ephedrine, norepinephrine, and *m*-synephrine due to some structural similarities [19–24]. However, this assumption is not borne out based on the receptor binding studies discussed below which indicate that *p*-synephrine exhibits little binding affinity for *α*- as well as *β*-1 and *β*-2 adrenoreceptors. 

**Figure 8:**
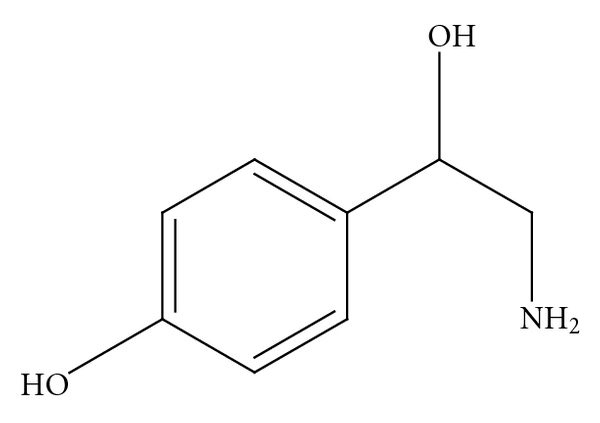
*p*-Octopamine.

 Based on receptor binding studies using human and animal cells, *m*-synephrine exerts its effects on *α*-  , *β*-1 and *β*-2 adrenergic receptors [28–32] resulting in increased blood pressure and heart rate effects. Jordan et al. [28] compared the receptor binding activities of *m*- and *p*-synephrine and *m*- and *p*-octopamine (N-demethyl *p*-synephrine; Figure 8) with norepinephrine in guinea pig atria and trachea [28]. *p*-synephrine represents over 90% of the protoalkaloids in bitter orange extract [12, 13], while *p*-octopamine is a minor constituent and is absent in many *Citrus* species [4]. The *meta*isomers do not occur in bitter orange extract [1–15]. In this study [28], *m*-synephrine was found to be 100-fold and *p*-synephrine 40,000-fold less potent than norepinephrine while the two octopamine isomers were about 6000-fold less potent than norepinephrine with respect to binding to *β*-1 and *β*-2 adrenoreceptors. The results indicate that small but crucial structural differences translate into markedly different receptor binding characteristics.

 The binding affinities of *p*-synephrine, *m*-synephrine, *β*-phenethylamine, and nor-ephedrine were compared by Ma et al. [29] on selected human *α*-adrenoreceptor subtypes. With respect to human *α*-1a adrenoreceptors, *p*-synephrine gave a maximal response at 100 *μ*M that was equal to 55% of the *m*-synephrine maximum. However, this concentration of *p*-synephrine is approximately 8,000-fold greater than the blood levels (2 ng/mL) obtained when a dose of 46.9 mg *p*-synephrine is consumed orally and has little or no effect on heart rate or blood pressure [33]. In addition, the presence of the *para*hydroxyl on *p*-synephrine reduced the receptor binding with respect to human *α*-2a and *α*-2c adrenoreceptors [29]. The authors concluded that *p*-synephrine may act as an antagonist rather than an agonist of presynaptic *α*-2a and *α*-2c adrenoreceptors present in nerve terminals [29]. Therefore, *p*-synephrine would not be expected to produce significant vasoconstriction as a result of binding to these receptors.

Brown et al. [30] compared the binding activities of the *meta*- and *para*isomers of synephrine and octopamine to rat aorta *α*-1 and rabbit saphenous vein *α*-2 adrenoreceptors. The binding of *m*-synephrine to *α*-1 and *α*-2 adrenoreceptors was 6-fold and 150-fold less, respectively, than norepinephrine. Furthermore, *p*-synephrine and p-octopamine were 1000-fold less active than norepinephrine in binding to these two receptors, again demonstrating that *m*-synephrine much more actively binds to these receptors than *p*-synephrine. Based on these receptor binding studies, *p*-synephrine would be expected to exert little vasoconstriction and, therefore, little or no effect on blood pressure as compared to *m*-synephrine or norepinephrine.

Hwa and Perez [31] examined the structural features necessary for binding of ligands to the *α*-1a adrenoreceptor and its subsequent activation. They concluded that it is the *meta*hydroxy of *m*-synephrine that hydrogen bonds to a specific serine moiety at the receptor site as opposed to the *para*hydroxy of *p*-synephrine. Thus, based on this study, *m*-synephrine (phenylephrine) binds and activates the receptor resulting in vasoconstriction and an increase in blood pressure, while *p*-synephrine does not.

Hibino et al. [32] examined the ability of *p*-synephrine to constrict isolated rat aorta at concentrations of 1 × 10^−7^ to 3 × 10^−5^ M. Using selected receptor antagonists, the authors concluded that the constrictor effects were exerted via *α*-1 adrenoreceptors and serotonergic (5-HT_1D _ and 5-HT_2A_) receptors. Again, the concentrations of *p*-synephrine used to produce aortic constriction were significantly higher than the peak blood levels (2 ng/mL) observed when 46.9 mg *p*-synephrine was given orally to human subjects and exerted little or no effect on heart rate or blood pressures [33]. For example, a concentration of 1 × 10^−6^ M is approximately 80-fold higher than the blood levels obtained in the above study with orally administered *p*-synephrine [3] at a dose of *p-*synephrine that is at the high end of doses commonly used in oral preparations for weight management and sports performance. These results suggest that* p*-synephrine may produce vasoconstriction but only at concentrations many times above the blood levels achieved under normal conditions of oral usage.

Rossato et al. [34] examined the uptake of *p*-synephrine and *m*-synephrine by rat cardiomyocytes at 1 mM concentrations and the effects of these two isomers on intracellular glutathione content. Both isomers were taken up by the cells with only the *m*-synephrine resulting in a depletion of both total and reduced glutathione. The effect was independent of *α*-1 adrenoreceptor stimulation since prazosin was not able to alter *m*-synephrine-induced glutathione depletion. The concentration of the two isomers that was used was over 80,000-fold higher than the 2 ng/mL peak blood levels observed following oral ingestion of 46.9 mg *p*-synephrine [33]. Thus, the clinical relevance of the results is questionable. However, the study does demonstrate that the cardiomyocytes differentiate between the two isomers, and only the *meta*isomer produced a potentially toxic effect.

Activation of *β*-3 adrenoreceptors has been shown to reduce food intake and weight gain in rats as well as enhance lipolysis in adipose tissue, while improving insulin resistance, glycemic control, and lipid profiles [35–38]. *β*-3 adrenoreceptors endogenously exist in white and brown adipose tissues and muscle, and activation increases lipolysis and lipid metabolism [35, 37, 38]. Increased expression of *β*-3 adrenoreceptor mRNA was shown to be associated with lower fasting plasma glucose, insulin, leptin, adiponectin, and lipids in obese Zucker rats [36]. Furthermore, studies indicate that *β*-3 adrenoreceptor-mediated lipolysis in adipocytes is mediated via the adenyl cyclase/cAMP/protein kinase A signaling cascade with downstream production of nitric oxide (NO). Thus, *p*-synephrine may act as a *β*-3 adrenoreceptor agonist, resulting in increased metabolic rate and lipolysis [27, 35, 39–42] as well as exhibiting hypoglycemic and insulin stimulatory properties [43, 44]. In contrast, Shannon et al. [45] have shown that epinephrine (adrenaline) lacks binding to human *β*-3 adrenoreceptors while its thermogenic effects result from direct *β*-1/2 adrenoreceptor binding.

Carpene et al. [41] assessed the lipolytic action of various potential *β*-3 adrenoreceptor agonists in white fat cells from humans, rats, hamsters, dogs, and guinea pigs. *p*-synephrine was partially active in stimulating lipolysis in all species while dopamine, tyramine, and *β*-phenylethylamine were ineffective. * p*-octopamine was shown to be the most selective for *β*-3 adrenoreceptors. In a Chinese hamster ovary cell line expressing *β*-3 adrenoreceptors, *p*-octopamine exhibited binding with only twofold less affinity than norepinephrine. 

Tsujita and Takaku [42] have shown that a segment wall extract rich in *p*-synephrine from mandarin oranges induced lipolysis in rat fat cells in a concentration-dependent manner. A juice sac extract from this same source failed to induce lipolysis. The nonselective *β*-antagonist propranolol completely inhibited lipolysis, indicating the involvement of a *β*-adrenoreceptor. The *α*-antagonist phenoxybenzamine had no effect on lipolysis indicating a lack of involvement of *α*-adrenoreceptors. These studies provide additional mechanistic insight into the thermogenic and potential weight management effects of* p*-synephrine. 

Parmar and Kar [43, 44] have examined the effects of a *Citrus sinensis* peel extract on various metabolic parameters in rats and mice. At a dose of 25 mg/kg, the extract exhibited hypoglycemic, insulin stimulatory, antiperoxidative, antithyroidal, and cardioprotective properties and decreased the concentrations of cholesterol and triglycerides in serum after daily dosing for 10 days. Decreases in lipid peroxidation were observed in heart, liver, and kidneys of rats [43]. These responses are consistent with *β*-3 adrenoreceptor activation [35–38]. However, exhibited hypoglycemic, insulin stimulatory, antiperoxidative, antithyroidal, and cardioprotective properties and decreased the concentrations of cholesterol and triglycerides in serum, the authors did not determine the content of* p*-synephrine or other constituents in the extracts, nor did they assess *β*-3 adrenoreceptor binding.

The above studies by Parmar and Kar [43, 44] are consistent with the results of Arbo et al. [46] who treated mice daily with bitter orange extract (7.5% *p-*synephrine) at doses of 400, 2,000, or 4,000 mg/kg (corresponding to 30, 150, and 300 mg *p-*synephrine/kg) or 30 or 300 mg *p*-synephrine/kg. A reduction in body weight gain was observed at all doses, while no effects were observed on organ weights, biochemical parameters, blood pressure, or heart rate in the treated mice relative to controls. Both doses of *p*-synephrine and the high dose of the bitter orange extract increased hepatic-reduced glutathione, while bitter orange extract decreased lipid peroxidation, and *p*-synephrine increased the antioxidant enzyme catalase by 6-fold. These antioxidant and tissue-protective properties of bitter orange extract and *p*-synephrine are consistent with the widely demonstrated beneficial effects of plant polyphenols [47, 48]. However, it should be noted that a dose of *p*-synephrine at 300 mg/kg is up to 600× doses commonly used in humans. Hansen et al. [49] have reported that a daily dose of *p*-synephrine of 100 mg/kg given to pregnant rats does not produce maternal or developmental toxicity.

Mercader et al. [50] compared the lipolytic activity of *p*-synephrine, *p*-octopamine, tyramine, and N-methyltyramine as well as the synthetic product isopropyl-norsynephrine in rat and human adipocytes against the prototypical *β*-adrenoreceptor agonist isoprenaline. *p*-octopamine (Figure 8) was slightly more active at stimulating lipolytic activity than *p*-synephrine, while tyramine and N-methyltyramine exhibited modest inhibitory activity. *p*-synephrine did not amplify the *β*-adrenergic component of epinephrine (adrenaline). Furthermore, *p*-synephrine was unable to block *α*-2 adrenoreceptors and did not provoke noticeable stimulation of glucose transport suggesting an impairment of insulin-activated glucose transport. These results strongly suggest but do not prove the involvement of *β*-3 adrenoreceptors. It can be concluded that with respect to bitter orange extracts the higher the amount of *p*-synephrine and the lower the amounts of tyramine and N-methyltyramine the more the lipolytic activity. As previously noted, *p*-octopamine is either absent or present in very minor amounts in bitter orange extracts [1–15].

In recent years, *β*-3 adrenoreceptors have been identified in cardiovascular tissues, and this observation has challenged the classical paradigm of sympathetic regulation by *β*-1 and *β*-2 adrenoreceptors [51, 52]. Evidence suggests that activation of cardiovascular *β*-3 adrenoreceptors produces a negative inotropic effect that antagonizes *β*-1 and *β*-2 adrenoreceptor activity, resulting in the modulation of sympathetic overstimulation [51]. The mechanism of action of this *β*-3 adrenoreceptor effect is believed to occur through regulation of nitric oxide [51]. Thus, one can postulate that *p*-synephrine stimulation of *β*-3 adrenoreceptors in the cardiovascular system will not result in an increase in blood pressure or heart rate but may exhibit a cardioprotective effect. This cardiovascular receptor response may explain why an increase in heart rate or blood pressure is generally not seen when *p*-synephrine is used alone or when caffeine is combined with *p*-synephrine or bitter orange extract in dietary supplements, in spite of the fact that caffeine alone is well known to affect these parameters [53], particularly in caffeine-sensitive individuals [54].

It is worth noting that octopamine receptors are present in insects, mollusks, and other invertebrates and are believed to represent counterparts of adrenoreceptors in vertebrates [55, 56]. Octopamine functions as a neuromodulator, neurotransmitter, and neurohormone in the nervous systems of invertebrates [56]. Both *α*- and *β*-adrenoreceptor-like octopamine receptor subtypes have been observed and studied [55, 57–59]. However, octopamine receptors differ markedly from mammalian adrenoreceptors in their ligand binding characteristics. For example, *p*-synephrine has been shown to have a binding affinity similar to octopamine for octopamine receptors [60–62], while epinephrine, norepinephrine, tyramine, phenylethylamine, phenylethanolamine, and dopamine either have no effect or moderate effect [61, 62]. Thus, the relative binding affinities for octopamine receptors are vastly different from the binding affinities and effects produced by these same ligands with respect to mammalian adrenoreceptors, and as a consequence, one cannot meaningfully extrapolate results from one system to the other. 

In summary, the binding studies discussed above provide extensive evidence that the *meta*- and *para*isomers of synephrine do not exhibit similar receptor binding and provide a mechanistic understanding for the vastly different pharmacological effects of the two isomers. The assumption that the two forms of synephrine have similar if not identical effects has lead to misinformation and the inappropriate attribution of potentially adverse effects produced by *m*-synephrine to *p*-synephrine, with only the latter occurring naturally in plant materials included in bitter orange [26]. Furthermore, structural differences and the resultant altered receptor-binding affinities also explain the markedly differing pharmacological responses to similar concentrations and doses of *p*-synephrine relative to ephedrine, norepinephrine, and other biogenic amines.

## 3. Observed Cardiovascular Effects

The receptor binding studies discussed above provide a mechanistic understanding for the observed effects of *p*-synephrine in human clinical studies as well as in animals. The vast majority of human clinical studies have noted that *p-*synephrine and bitter orange extract either alone or in combination with caffeine and other ingredients have no effect on blood pressure [19, 20, 63–71] or heart rate [19, 33, 63–71], in spite of the widely postulated assumption that these effects would occur [19–23, 72–74]. Animal studies involving orally administered *p*-synephrine and bitter orange extract have similarly demonstrated the absence of cardiovascular affects [34, 75–77], consistent with the poor binding affinity of *p*-synephrine for *α*- as well as *β*-1 and *β*-2 adrenoreceptors. Huang et al. [77] have shown that at a dose of 1 mg/kg orally twice a day in two models of portal hypertensive rats, *p*-synephrine significantly ameliorated the hyperdynamic effects. 

Several human studies involving products that contained *p-*synephrine in addition to other constituents including caffeine have reported increases in blood pressure [71, 78] and heart rate [33, 71], and the authors have suggested that these effects may be due to* p*-synephrine. The study by Haller et al. [33] demonstrated that a single oral dose of *p*-synephrine alone (46.9 mg, as 6% *p*-synephrine in a bitter orange extract, Advantra Z, Nutratech Inc., West Caldwell, NJ, USA) had no effect on systolic or diastolic blood pressure, while an increase in heart rate occurred after six hours. These results are complicated by the fact that the subjects consumed a meal three hours after receiving the *p*-synephrine, and the thermic and cardiovascular effects of food have been demonstrated in other studies [66]. In addition, the half-life of *p-*synephrine is two to three hours [33, 71, 79], and as a consequence, it is doubtful that one would expect to see a significant cardiovascular effect after two to three half-lives when none occurred at earlier time points.

 Another study reported small, statistically significant but clinically insignificant increases in heart rate and blood pressure [78] following oral administration of a bitter orange extract preparation reported to contain 54 mg *p*-synephrine. However, a study by Min et al. [64] using the same product in a similarly designed randomized, placebo-controlled crossover study observed no effect on systolic or diastolic blood pressure or on the rate-corrected QT (QTc) interval. One eight-week placebo-controlled double-blind study has reported a small but significant decrease in systolic and diastolic blood pressures following ingestion of a product containing a *Citrus* peel extract [80]. A significant loss of weight as well as decreases in fasting glucose, triglycerides, and cholesterol were also observed. 

McGuffin [16] reviewed the FDA adverse events reports concerning bitter orange prior to 2004 and concluded that no adverse events could be attributed directly to bitter orange extract or *p*-synephrine. Subsequently, Stohs [17] reviewed and assessed the 22 FDA adverse event reports (AERs) from April 2004 through October 2009 associated with bitter-orange-containing products, as well as 10 clinical case reports published during this time interval which purported to link bitter-orange-containing weight management products with cardiovascular incidents and other adverse events. In each case, the authors implicated bitter-orange extract and/or *p*-synephrine as the possible causative agent. However, in all AERs and case reports, the products involved were polyherbal, polyalkaloidal and poly-protoalkaloidal, and as a consequence, it is not possible or plausible to ascribe the observed effects to a single ingredient. 

 A wide range of confounding factors existed among the published case reports including a history of obesity, heart murmur, preexisting heart disease, hypertriglyceridemia, sickle cell trait, dehydration, pneumonia, smoking, physical inactivity, possible use of anabolic steroids and/or performance enhancing drugs, gastroesophageal disease, high caffeine intake, and high alcohol consumption [17]. In addition, products were not always being taken as recommended, and it was not always clear if the subjects were using other unreported dietary supplements and/or drugs. The high caffeine intake associated with the products in question may have also been a contributing factor [17]. The observed effects may have been due to a combination of ingredients. Finally, the possibility exists that the consumption of the product and the adverse event were concurrent but unrelated since millions of individuals use *p*-synephrine-containing dietary supplements and food products on a daily basis while millions of cardiovascular events occur annually. 

In summary, the results of human clinical and animal studies involving *p*-synephrine are consistent with the results of adrenoreceptor binding studies. The observed lack of cardiovascular effects of orally administered *p*-synephrine is in concert with its poor binding affinity for *α*- as well as *β*-1 and *β*-2 adrenoreceptors.

## 4. Weight Loss and Weight Management Effects

If *p-*synephrine binds to and activates *β*-3 adrenoreceptors, an increase in thermogenesis and lipolysis should be expected. Various animal studies have shown that the oral administration of *p*-synephrine or *p-*synephrine in the form of bitter orange extract results in weight loss or decreased weight gain [46, 75, 76]. The thermic effect of orally administered *p*-synephrine (26 mg) was demonstrated in a five-hour human clinical study involving 30 subjects [66]. Stohs et al. [69] have recently shown that the oral administration of 50 mg *p*-synephrine (30% *p*-synephrine as Advantra Z) increased resting metabolic rate with no effect on heart rate or blood pressure in humans in a double-blinded, randomized placebo-controlled study. 

*p*-synephrine in the form of bitter orange extract is widely used in conjunction with other ingredients in products designed to support weight loss/weight management and/or enhance sports performance. Both published and unpublished clinical studies have been extensively reviewed by Stohs and Shara [81, 82], and various studies have demonstrated modest weight loss. However, since *p*-synephrine is combined with other ingredients as caffeine and various herbal constituents, it is not possible to directly attribute the thermogenic and weight loss effects of the products to *p*-synephrine. Unfortunately, the effect of *p*-synephrine alone on weight loss in a long-term human study has not been assessed.

The effect of a performance-enhancing dietary supplement containing *p*-synephrine was examined under resting and exercise conditions [71]. Exercise was perceived as being less strenuous after consumption of the product. However, the product contained *p*-synephrine as well as other herbal ingredients, and as a consequence, the effects cannot be specifically ascribed to *p*-synephrine.

## 5. Summary and Conclusions

Receptor binding studies involving tissues from humans and animals indicate that *p*-synephrine exhibits poor binding affinities for *α*-adrenoreceptor subtypes as well as *β*-1 and *β*-2 adrenoreceptors relative to norepinephrine, *m*-synephrine, ephedrine, and other amines. Activation of these receptors is associated with increased vasoconstriction, cardiovascular constriction, and heart rate. Studies in animals and humans have demonstrated that *p*-synephrine exhibits little or no cardiovascular activity when administered orally either alone or in the form of products containing multiple ingredients. 

A number of case reports have implied that *p*-synephrine and/or bitter orange extract may have been responsible for adverse cardiovascular and other events. However, in each case, the product presumably consumed was polyherbal as well as polyalkaloidal and poly-protoalkaloidal, and as such, the adverse effect involved cannot be directly ascribed to *p*-synephrine. Furthermore, various confounding factors were involved, and a number of plausible explanations exist for the observed effects. Several recent investigations involving the administration of* p*-synephrine to human subjects at doses higher than those present in the clinical case reports have not observed cardiovascular effects. 

Several studies indicate that *p*-synephrine may bind to *β*-3 adrenoreceptors which is associated with thermogenesis, lipolysis, glucose as well as cholesterol metabolism, and possibly reduced food intake. More definitive studies involving *p*-synephrine and *β*-3 adrenoreceptors are needed. Unfortunately, an appropriate ligand for the *β*-3 adrenoreceptor currently does not exist. However, measuring a functional response such as cyclic AMP elevation in a human adipocyte cell line that expresses the *β*-3 adrenoreceptor, and assessing potencies of antagonists to block this response may be appropriate. Several animal studies have clearly shown that oral *p*-synephrine administration can support weight loss and/or reduced weight gain without adverse cardiovascular effects. A number of human clinical studies involving oral administration of multi-ingredient products containing *p-*synephrine have demonstrated modest weight loss, although weight loss has not occurred in all cases. No human study involving the long-term administration of* p*-synephrine alone has been conducted with respect to weight loss and weight management, and the need for this study is apparent.

*p*-synephrine has been shown to bind to octopamine receptor subtypes in invertebrates. These receptors are believed to be analogous to adrenoreceptors in vertebrates. However, the binding affinities of various amines including *p*-synephrine, *m*-synephrine, and norepinephrine to octopamine receptors are markedly different and clearly unrelated to their binding characteristics to adrenoreceptors. As a consequence, the binding of *p*-synephrine to octopamine receptors cannot be extrapolated to adrenoreceptors in humans and other vertebrates.

In general, additional human studies are required involving long-term safety and weight loss where *p*-synephrine is administered alone and/or with a small number of selected and clearly defined additional ingredients as opposed to being included in complex multiherbal products. Furthermore, additional *p*-synephrine adrenoreceptor binding studies are needed, in particular, involving *β*-3 adrenoreceptors in order to provide greater clarity with respect to both safety and efficacy.
